# Case report: lipoprotein apheresis reduces the risk of cardiovascular events and prolongs pregnancy in a woman with severely elevated lipoprotein(a), cardiovascular disease, and a high risk of preeclampsia

**DOI:** 10.3389/fmed.2023.1190446

**Published:** 2023-09-20

**Authors:** Joanna Marlȩga-Linert, Katarzyna Wartecka-Zielińska, Dariusz Wydra, Marcin Fijałkowski, Marcin Gruchała, Agnieszka Mickiewicz

**Affiliations:** ^1^First Department of Cardiology, Medical University of Gdańsk, Gdańsk, Poland; ^2^Department of Gynecology and Obstetrics, Medical University of Gdańsk, Gdańsk, Poland

**Keywords:** pregnancy, preeclampsia, lipoprotein (a), lipoprotein apheresis, case report

## Abstract

**Background:**

Preeclampsia is a common and serious pregnancy-induced disease, with potential severe maternal and fetal complications. Recently, an increased lipoprotein (a) (Lp[a]) concentration, an important factor in cardiovascular diseases (CVDs) pathogenesis, has been identified as a sensitive and specific marker of preeclampsia severity. Although lipoprotein apheresis (LA) is currently used in patients with hyperlipoproteinemia(a) and CVD, real-life data on its efficacy among pregnant women with an increased risk of preeclampsia are limited.

**Case presentation:**

We present the case of a pregnant woman with severely elevated Lp(a), two previous episodes of the acute coronary syndrome and multivessel coronary disease treated with long-term LA before pregnancy, and a high risk of preeclampsia (as assessed using combined test screening). An increased pulsatility index and early diastolic notch were observed on Doppler interrogation at 18 weeks’ gestation. Biweekly LA therapy was re-initiated at 21 weeks’ gestation. The LA safely removed 70% of the serum Lp(a) concentration and reduced low-density lipoprotein-cholesterol (LDL-C) levels by 60%. We also observed an improvement in her urine protein/creatinine ratio, a reduction in the pulsatility index, and a notch on Doppler interrogation. The pregnancy lasted until week 36, when severe preeclampsia prompted an emergency cesarean delivery.

**Conclusion:**

Pregnancy in women with elevated Lp(a), CVD, and a high risk of preeclampsia can present challenges in clinical management. Our case report indicates the benefits of LA in preventing atherosclerotic CVD progression during pregnancy, its potential influence on uteroplacental circulation, and prolongation of pregnancy for the best possible intrauterine fetus development. LA may be considered as a treatment option during pregnancy in such conditions. In addition, in pregnant women with CVD, we suggest screening using a combined test and measurement of Lp(a) as a marker of preeclampsia severity.

## 1. Introduction

Preeclampsia (PE) is a serious pregnancy-induced disease with potential severe maternal and fetal complications. It is caused by the improper invasion of the myometrium by the spiral arteries, which begins in the early stages of gestation. High blood pressure and proteinuria are the most common symptoms. However, the complexity of the disease results in diverse manifestations, including maternal renal and liver damage, maternal neurological and hematological symptoms, placental malfunction, fetal growth retardation, and preterm delivery. Ideally, high-risk individuals should be identified and administered with pharmacological prophylaxis. Severe PE requires urgent delivery to save the lives of the mother and her child. However, early PE is more challenging to manage, because termination of pregnancy is very hazardous due to risk of infant prematurity and associated complications. The recommended management strategy of early PE is one that safely enables prolongation of pregnancy and the best possible intrauterine fetal development (1, 2).

Recent studies have indicated similarities in the pathophysiologies of PE and cardiovascular disease (CVD). An increased lipoprotein(a) [Lp(a)] concentration has been proven to be an important risk factor for both conditions (3, 4). Lipoprotein apheresis (LA) is currently recommended for treatment of patients with hyperlipoproteinemia(a) [hyper-Lp(a)] and CVD, since pharmacotherapy is still undergoing investigation in clinical trials (3, 5–12). LA has been also investigated as an emerging treatment option in PE (13–15). Nevertheless, there remains a lack of data on LA therapy among pregnant women with an increased risk of PE (16).

Pregnant women with elevated Lp(a), CVD, and a high risk of PE may be challenging to manage. Thus, we describe the case of a pregnant woman with advanced, premature atherosclerotic CVD (ASCVD); severe hyper-Lp(a); and a high risk of PE who was treated with LA, since 21 weeks of gestation with a good clinical effect. To our knowledge, this is the first report on LA therapy for this clinical condition and could be serve as evidence in support of the safety and efficacy of LA during pregnancy.

## 2. Case description

A 37-year-old woman with advanced, progressive, premature ASCVD and severe hyper-Lp(a) was admitted to the Lipoprotein Apheresis Unit at the First Department of Cardiology in Gdansk. She had a past medical history of non-ST-elevation myocardial infarction, which was treated with a two-staged percutaneous coronary intervention of the right coronary and circumflex arteries and implantation of two drug-eluting stents. This was followed by an incident of unstable angina with left anterior descending artery angioplasty and additional drug-eluting stent implantation. Atheroma plaques in both the internal carotid arteries, with 50%–69% stenosis, were also confirmed.

The patient has none of the following cardiovascular risk factors: cigarette smoking, arterial hypertension, diabetes, and obesity. In addition, she did not present a clinical phenotype of familial hypercholesterolemia. The maximal untreated low-density lipoprotein cholesterol (LDL-C) level in this patient was 160 mg/dl and a family history of hypercholesterolemia was negative. However, her father had experienced myocardial infarction at 40 years of age, without having Lp(a) measured, which indicated a family history of premature CVD. She had a history of miscarriage and three unsuccessful *in vitro* fertilization (IVF) procedures. The most common causes of thrombophilia were excluded, namely Leiden mutation, antiphospholipid syndrome, mutation in the prothrombin gene, antithrombin deficiency, and protein C and S deficiency. However, a methylenetetrahydrofolate reductase C677 mutation and a plasminogen activator inhibitor type 1 (PAI1) 4G/4G homozygous mutation were confirmed.

The patient’s untreated LDL-C and Lp(a) levels were 160 mg/dL and 249 mg/dL, respectively. The corrected LDL-C level, calculated using Dahlén’s formula was 83 mg/dL {LDL-C_*corrDahl*é*n*_ = laboratory LDL-C−[Lp(a) mass × 0.30]} (17, 18). The patient was administered with 20 mg of rosuvastatin and 10 mg of ezetimibe daily, which reduced the LDL-C level to 54 mg/dL. A further increase in the rosuvastatin dose resulted in recurrent muscle pain.

Considering the severe hyper-Lp(a) and advanced premature CVD, the patient was initiated on regular biweekly LA after the myocardial infarction episode. Cascade filtration technique (MONET/Fresenius), a method of selective, extracorporeal removal of pro-atherosclerotic lipid particles was used. In MONET system after separation plasma is transferred through the second polysulphone filter, which allows albumin, HDL, and smaller immunoglobulins to pass, whereas Lp(a), LDL, VLDL and chylomicrons, are retained. LA-induced median Lp(a) reduction prior to the pregnancy was 65% (IQR 58–71). After 2 years of regular LA, the patient underwent an IVF procedure with pre-implantation genetic diagnostics. Directly before embryo transfer in September 2021, the patient stopped taking statins. Weighting the risk of potential uterine bleeding and loss of pregnancy, and the risk of CVE, shared decision-making brought us to suspending LA procedures after confirmation of pregnancy. The IVF procedure was successful, and she became pregnant. The patient was treated with subcutaneous enoxaparin (60 mg) and continued aspirin (75 mg) daily. Owing to a diagnosis of gestational diabetes at 8 weeks’ gestation, a long-acting insulin analog therapy was initiated at 8 units and gradually increased during the pregnancy.

## 3. Diagnostic assessment

At 12 weeks’ gestation, fetal ultrasonography and pregnancy-associated plasma protein A (PAPP-A) test results indicated a high risk of PE and intrauterine fetal growth restriction (FGR) (Table 1). The patient’s aspirin dose was increased to 150 mg daily, and regular uterine Doppler ultrasound examinations and urinalyses were scheduled. Repetitive 24 h ambulatory and home blood pressure monitoring indicated values in the expected range. A routine check-up between 17–18 weeks’ gestation revealed elevated LDL-C and triglyceride serum concentrations of up to 191 mg/dL and 179 mg/dL, respectively. Fetal ultrasonography revealed an increased pulsatility index (PI) and early diastolic notch on Doppler interrogation. To decrease the risk of both, PE development and progression of ASCVD, patient restarted regular biweekly LA at 21 weeks’ gestation. Patient underwent seven LA procedures during pregnancy (Table 2). LA procedures lasted 270 ± 40 min. Heparin in a dose of 5000 IU in priming solution was used, followed by the acid citrate dextrose anticoagulation in a ratio 1:20 – 1:40. Plasma volume of 3,200 ± 705 ml was treated from venous access without any significant adverse events. During pregnancy we aimed to increase a volume of plasma treated up to 3,600 ml (from 3,000 ml before pregnancy). Before and after each LA procedure, blood and urine tests were performed, which included tests for lipid parameters, urine protein/creatinine ratio (UPCR), and urinary protein excretion. Both the UPCR and urinary protein excretion normalized after a single LA procedure (Table 2). Furthermore, we noted a reduction in the PI and notch on Doppler interrogation.

**TABLE 1:** Combined screening at 12 weeks’ gestation.

Biochemic parameters and condition tested	Result and risk
Beta subunit of human chorionic gonadotropin (hCG beta)	19.1 IU/I (0.486 MoM)
Preeclampsia before 34 weeks’ gestation	1:107
Preeclampsia before 37 weeks’ gestation	1:35
Intrauterine fetal growth restriction before 37 weeks’ gestation	1:97

**TABLE 2:** Biochemical parameters before and after LA.

	NR	Pre-P	17WG	21WG, pre-LA1	21WG, post-LA1	23WG, pre-LA2	23WG, post-LA2	25WG, pre-LA3	25WG, post-LA3	27WG, pre-LA4	27WG, post-LA4	29WG, pre-LA5	29WG, post-LA5	31WG, pre-LA6	31WG, post-LA6	33WG, pre-LA7	33WG, post-LA7
UPCR (mg/g)	<150	ND	ND	ND	ND	185.55	91.54	ND	ND	122.10	ND	162.19	ND	ND	ND	256.38	ND
UPE (G/L)	<0.15	ND	ND	ND	ND	0.32	0.07	<0.07	ND	0.08	ND	0.11	ND	<0.07	ND	0.26	ND
Lp(a) (mg/dl)	<30	22	170	170	ND	170	52	ND	48	148	ND	158	52	124	42	100	33
TC (mg/dL)	<190	286	314	210	147	288	139	329	146	298	ND	297	135	303	135	ND	127
LDL-C (mg/dL)	<55	ND	191	129	72	169	66	207	69	171	ND	172	52	189	66	ND	53
HDL-C (mg/dL)	>45	43	87	54	ND	80	56	89	57	88	ND	84	59	74	50	ND	52
TG (mg/dL)	<150	360	179	193	103	194	87	226	101	194	ND	205	119	316	123	ND	140

HDL-C, high-density lipoprotein cholesterol; LA, lipoprotein apheresis; LDL-C, low-density lipoprotein cholesterol; ND, no data, NR, normal range; Pre-P, pre-pregnancy; TC, total cholesterol, TG- triglicerydes; UPE, urinary protein excretion; UPCR, urine protein/creatinine ratio; WG, weeks of gestation.

Blood pressure and urine dipstick monitoring at home revealed normal results. However, at 33 weeks’ gestation, an increase in the blood pressure was noted (mean home blood pressure, 152/98 mmHg). Methyldopa was immediately administered in adjusted doses; however, a further increase in the blood pressure was observed over 16 days.

## 4. Therapeutic intervention

At 36 weeks’ gestation, the patient required admission to the obstetrics and gynecology clinic for severe PE with uncontrolled blood pressure and severe proteinuria (10 g/24 h). After 3 days of hospitalization, a successful urgent cesarean delivery was performed under general anesthesia. The female infant weighed 1,890 g, which is below the 10th centile for gestational age. Due to a difficult extraction attributed to the transverse lie, prematurity, FGR, and general anesthesia, the newborn was assessed to have a low Apgar score (1, 6, 8, and 9 points at 1, 3, 5, and 10 min, respectively); however, upon further observation, the infant showed no neurological deficits.

Sudden maternal thrombocytopenia was observed after the cesarean section (platelet count, 50–60 G/L). The detection of antibodies to platelet factor 4 complexes and a suspicion of heparin-induced thrombocytopenia resulted in a conversion to fondaparinux. Moreover, significant increases in the alanine transaminase (87 U/L), aspartate transaminase (83 U/L), and lactate dehydrogenase (564 U/L) levels were observed. The patient’s hemoglobin level decreased to 7.6 g/dL, although no significant blood loss was observed. This triad of symptoms indicated the development of the “hemolysis, elevated liver enzymes, and low platelets” (HELLP) syndrome. Oliguria and severe systemic edema occurred due to worsening renal function (glomerular filtration rate, 44 mL/min). Intravenous diuretics and albumin supplementation normalized both clinical and laboratory parameters. The administration of statins and ezetimibe was immediately restarted after delivery.

Due to a slight elevation in the troponin level and transient chest pain, the patient underwent echocardiography and Holter electrocardiogram, which did not reveal any abnormalities. Doppler ultrasonography of the deep veins of the lower limbs excluded thrombosis. Based on the overall clinical presentation and diagnostic results, a pulmonary embolism was excluded, and type 2 acute coronary syndrome was suspected. However, owing to the resolution of clinical and laboratory symptoms and a lack of significant deviations in diagnostic imaging, angiography was waived.

## 5. Follow-up and outcomes

After 13 days of hospitalization, the patient was discharged with a restarted statin regimen (rosuvastatin 20 mg daily) in combination with ezetimibe 10 mg daily, followed by LA 5 weeks later. Coronary computed tomography angiography performed 3 months after delivery did not show any changes in the extent of the patient’s coronary artery disease during the pregnancy. Pre-apheresis LDL-C level on lipid lowering medications 5 weeks after delivery was 34 mg/dl. Table 3 shows a timeline with relevant data from this episode of care. This case report was specifically discussed with the patient, and informed consent to publish was obtained.

**TABLE 3:** Timeline with relevant data from this episode of care.

Timeline
***In vitro* fertilization (IVF) procedure**
↓
8 weeks’ gestation: gestational diabetes treated with long-acting insulin analog therapy
↓
12 weeks’ gestation: fetal ultrasonography and pregnancy-associated plasma protein A test results revealed a high risk of preeclampsia (PE) and intrauterine fetal growth restriction
↓
17–18 weeks’ gestation: routine check-up between 17 and 18 weeks of gestation revealed elevated low-density lipoprotein cholesterol and triglyceride serum concentrations of up to 191 mg/dL and 179 mg/dL, respectively. Fetal ultrasonography revealed an increased pulsatility index and early diastolic notch on Doppler interrogation.
↓
21 weeks’ gestation: the patient was initiated on regular biweekly lipoprotein apheresis
↓
33 weeks’ gestation: an increase in the blood pressure was noted. Methyldopa was immediately administered in adjusted doses.
↓
36 weeks’ gestation: the patient required admission to the obstetrics and gynecology clinic for severe preeclampsia with uncontrolled blood pressure and severe proteinuria.
↓
After 3 days of hospitalization, a successful urgent cesarean delivery was performed under general anesthesia
↓
Sudden maternal thrombocytopenia was noted after the cesarean section (platelet count, 50–60 G/L). Detection of antibodies to platelet factor 4 complexes and a suspicion of heparin-induced thrombocytopenia led to a conversion to fondaparinux.
↓
Significant increases were noted in the alanine transaminase (87 U/L), aspartate transaminase (83 U/L), and lactate dehydrogenase (564 U/L) levels. The patient’s hemoglobin level decreased to 7.6 g/dL, although no significant blood loss was observed. This triad of symptoms indicated the development of the HELLP syndrome.
↓
Oliguria and severe systemic edema occurred due to worsening renal function (glomerular filtration rate, 44 mL/min). Intravenous diuretics and albumin supplementation normalized both clinical and laboratory parameters.
↓
The administration of statins and ezetimibe was immediately restarted after delivery.
↓
Slight elevation of the patient’s troponin level and transient chest pain were noted.
↓
Echocardiography and Holter electrocardiogram did not reveal any abnormalities. Doppler ultrasonography of the deep veins of the lower limbs excluded thrombosis.
↓
A pulmonary embolism was excluded and type 2 acute coronary syndrome was suspected.
↓
The patient was discharged after 13 days of hospitalization
↓
Coronary computed tomography angiography performed 3 months after delivery did not show any changes in the extent of the patient’s coronary artery disease during the pregnancy.

## 6. Discussion

Pregnancy in women with elevated Lp(a), CVD, and a high risk of PE can present challenges in the management of CVD progression and PE-related complications. Increased morbidity and mortality in a mother and a child have to be prevented (19). Considering that LA is recommended in severe lipid disorders, including elevated Lp(a) concentrations, along with the proven safety of regular LA therapy in pregnant women with homozygous familial hypercholesterolemia (HoFH), LA could be an option to safely treat hyperlipoproteinemia(a) during pregnancy (20–23). Although the American Society for Apheresis (ASFA) guidelines on the use of apheresis in clinical practice recommends LA in hyperlipoproteinemia(a) and ASCVD (grade II, 1B), there is no mention about LA therapy in pregnant women with hyper-Lp(a). The ASFA Guidelines referred also to apheresis use in preeclampsia, grading LA or therapeutic plasma exchange (TPE) for severe PE as category III (decision-making should be individualized), level of evidence 2C. The ASFA guidance referred to 19 studies of LA/TPE for PE but none of these were randomized controlled trials (RCTs) and only 4 were controlled trials (15). To our knowledge, this is the first report on LA therapy in a patient with this clinical condition and could support the safety and efficacy of LA during pregnancy.

In this case, LA safely removed 70% of the patient’s Lp(a) concentration and reduced 60% of her LDL-C level. Published data indicate that regular LA therapy results in an 80–92% reduction in the major adverse cardiovascular events. Furthermore, we observed a reduction in the UPCR and PI after LA, which indicated an improvement in uteroplacental circulation and an increased chance of prolonging the pregnancy (the main determinant of neonatal outcomes). The improvement in uteroplacental circulation could result from improved endothelium-dependent vasodilatation, as LA has been shown to present such an effect.

Obstetric societies recommend aspirin prophylaxis before 16 weeks’ gestation in all women with a history of PE, gestational hypertension, body mass index >30 kg/m^2^, and pre-gestational diabetes. Early screening for PE is also helpful in identifying high-risk women. Known predictors of PE at 11–13 weeks’ gestation are the mean arterial pressure, uterine artery PI, serum PAPP-A, placental growth factor, and soluble fms-like tyrosine kinase-1 (sFlt-1) (24). Our patient underwent screening using a combined test, and the risk of PE and FGR was assessed as high. Our patient was chronically on 75 mg of aspirin daily; this was immediately increased to 150 mg daily after her screening result. The recommended therapeutic option for patients at a moderate-to-high risk of PE is a daily dose of 100–150 mg of acetylsalicylic acid before 16 weeks’ gestation. Interestingly, aspirin also has the potential to reduce the Lp(a) concentrations by 20% and a greater decrease has been observed among patients with higher baseline Lp(a) levels above 30 mg/dL, irrespective of apo(a) isoform size (25). Lacaze et al. published the results of a study that indicated that aspirin decreased the rate of major adverse cardiovascular events in the primary prevention group (26).

Published data have indicated an association between hyperlipidemia and PE (19). The ABCD study showed that hypertriglyceridemia was related to PE (27). Li et al. showed that elevated triglyceride levels can be predictors of early onset PE (28). Another study, conducted among 50 women with a benign type of PE who were in their third trimester of pregnancy, revealed that Lp(a) was a predictor and marker of severe PE. An Lp(a) concentration of >40.5 mg/dL in a mildly pre-eclamptic patient was a predictor of the development of severe PE, and a concentration of >52.5 mg/dL was a sensitive and specific marker for PE severity. Lp(a) can enter arterial wall, stimulate endothelial activation and vascular wall inflammation (4, 29). Those findings suggest that Lp(a) is involved in PE pathogenesis (4, 16). As previously mentioned, the treatment of PE mainly includes hypotensive drugs and urgent delivery in the most severe cases (30). Hypolipemic drugs are generally not recommended for pregnant women, although some data have indicated their safety during pregnancy (31, 32). Döbert et al. analyzed the effectiveness of statin therapy among 1,120 women with a high risk of PE in the late stages of pregnancy (i.e., at 35–37 weeks’ gestation). Half of the patients were administered with pravastatin (20 mg daily), and the rest were assigned to the placebo group. The results showed no differences in the PE rates between the two groups (33).

In view of data indicating a relationship between hyperlipidemia and PE pathophysiology, LA was investigated as a therapeutic option for patients with severe and early PE. Numerous publications have shown that LA prolongs pregnancy in patients with PE; however, the underlying mechanism remains unclear (13). Clinical effect is obtained by the removal of atherogenic lipoproteins including Lp(a), but other factors have been also investigated. Thadhani et al. conducted dextran sulfate apheresis and investigated an elimination of sFlt-1 from 11 pregnant women (23–32 weeks’ gestation) with early PE. They found a 18% reduction in the sFlt-1 levels and an up to 44% reduction in the UPCR. Pregnancies were continued for a further 15 days in women who had undergone two or three apheresis procedures, compared with the 3 days in the women in the control group who did not undergo apheresis. Moreover, newborns of mothers who had undergone apheresis required lesser oxygen therapy and ventilation (34). Similar results were presented in “The Freiburg PE H.E.L.P.-Apheresis study.” Winkler et al. showed the safety and effectiveness of LA in pregnant women with PE. Pregnancies were prolonged, which allowed for the administration of steroids to induce fetal lung maturation. The authors reported that lowering the sFlt-1 level was crucial for the clinical benefits of LA in this condition (35). In our patient sFlt-1 levels were not measured due to inability to test it routinely in that time.

In the present case, the patient’s course of LA was safe without adverse effects, especially hypotension. Both the mother and the child were carefully monitored during the procedure. The pregnancy lasted for 36 weeks, which allowed the best possible intrauterine fetal development and avoided complications due to premature delivery. Careful monitoring also allowed an immediate reaction to the patient’s sudden development of severe PE with proteinuria and uncontrolled hypertension. A cesarean section was performed in the early phase of the HELLP syndrome, which was resolved with supportive treatment (36, 37).

To summarize, PE remains a poorly understood disease with complex pathophysiology and potentially serious maternal and fetal complications. Dyslipidemia, including hyper-Lp(a), may play a role in the pathogenesis of PE. Elevated Lp(a) levels can be a sensitive and specific marker of PE severity. Currently, there are no dedicated treatment methods for treating pregnant women with severely elevated Lp(a) levels, CVD, and a high risk of PE. Numerous studies have highlighted the beneficial effects of LA in PE and the safe prolongation of pregnancy.

Our case report indicates the safety and efficacy of LA during pregnancy in a patient with elevated Lp(a) and ASCVD, its potential influence on uteroplacental circulation, and the prolongation of pregnancy for the best possible intrauterine fetal development. We conclude that the early initiation of LA in our patient, before the onset of PE symptoms, improved endothelial dysfunction aggravated by the severely elevated Lp(a). No significant progression of ASCVD was noted in our patient. Further studies are needed to clarify, whether continuing LA from the point of conception could have improved an endothelial dysfunction and prevented the PE in patients on high risk of developing PE.

To our knowledge, this is the first case report on a pregnant woman with severely elevated Lp(a) levels and a high risk of PE who was safely and effectively managed with LA therapy. This report describes the usefulness of LA as a therapeutic option in pregnancy, highlights the importance of screening in the first trimester to evaluate the PE risk, and contributes to the literature on Lp(a) as both a cardiovascular risk factor and marker of PE severity.

## 7. Patient perspective

I and my husband were supposed to start training at the adoption center; however, I experienced myocardial infarction after a miscarriage and three unsuccessful IVF procedures. Shortly after the myocardial infarction episode, I was diagnosed with hyperlipoproteinemia (a) and was started on biweekly lipoprotein apheresis. While this new situation was terrifying, the knowledge that I was receiving effective treatment and that I was protected against disease progression reassured me. After 2 years of regular apheresis and intensive hypolipemic pharmacotherapy, we decided to undergo an IVF procedure for the final time. Although it required temporary suspension of the hypolipemic pharmacotherapy, I was ready to take the risk. Thankfully, this IVF procedure was successful, and I got pregnant. During pregnancy, I was under careful medical supervision, and both the cardiologist and gynecologist stayed in regular contact with me. As I was aware of the risks for both me and the baby, I complied with all the recommendations provided and participated in regular testing, such as attending the doctors’ appointments regularly and undergoing hypotensive drug adjustment, blood pressure measurements, and urine testing. I agreed to re-start the lipoprotein apheresis therapy at 21 weeks’ pregnancy to prevent cardiovascular disease progression and preeclampsia development. I was monitored carefully during the procedures, and no complications occurred. I believe that this management allowed sufficient time for my baby to mature and a prompt response while my condition worsened. After cesarean section, the hypolipemic drugs were adjusted immediately. I believe that I received an innovative treatment that allowed me to go through this pregnancy safely and deliver a healthy baby. Without a doubt, this was my only chance to realize my dream of having a child.

## Data availability statement

The original contributions presented in this study are included in the article/supplementary material, further inquiries can be directed to the corresponding author.

## Ethics statement

Ethical review and approval was not required for the study on human participants in accordance with the local legislation and institutional requirements. Written informed consent from the patient was not required to participate in this study in accordance with the national legislation and the institutional requirements. Written informed consent was obtained from the individual(s) for the publication of any potentially identifiable images or data included in this article.

## Author contributions

JM-L collected the blood, interpreted the data, and drafted the manuscript. AM arranged the medical treatment and drafted and finalized the manuscript. KW-Z, DW, MF, and MG contributed to the data analysis. All authors contributed to the interpretation of data and critical review of the manuscript and agreed to be held accountable for the content of the work.
